# Considerations for Public Health Organizations Attempting to Implement a Social Media Presence: A Qualitative Study

**DOI:** 10.2196/publichealth.5032

**Published:** 2016-02-24

**Authors:** Mark Hart, Nichole Stetten, Gail Castaneda

**Affiliations:** ^1^ University of Florida Gainesville, FL United States

**Keywords:** public health, social networking sites, professional development, training centers, Facebook, Twitter

## Abstract

**Background:**

In the past decade, social media has become an integral part of our everyday lives, but research on how this tool is used by public health workers and organizations is still developing. Budget cuts and staff reduction in county departments have required employees to take on more responsibilities. These reductions have caused a reduction in the time for training or collaborating with others in the field. To make up for the loss, many employees are seeking collaboration through social media sites but are unable to do so because state departments block these Internet sites.

**Objective:**

This study sought to highlight the key considerations and decision-making process for a public health organization deciding whether to implement a social media presence for their organization.

**Methods:**

Using 3 structured interviews, 15 stakeholders were questioned on their personal experience with social media, experience within the context of public health, and their thoughts on implementation for their center. Interviews were coded using constant comparative qualitative methods.

**Results:**

The following themes emerged from the interviews: (1) personal experience with technology and social networking sites, (2) use of social networking sites in public health, (3) use of social networking sites in work environments, (4) social networking sites access, (5) ways the Rural South Public Health Training Center could use social networking sites, and (6) perceived outcomes of social networking site usage for the Rural South Public Health Training Center (positive and negative).

**Conclusions:**

The collective voice of the center showed a positive perceived perception of social media implementation, with the benefits outweighing the risks. Despite the benefits, there is a cautious skepticism of the importance of social networking site use.

## Introduction

This research study was conducted with a focus on the Rural South Public Health Training Center (RSPHTC), a collaborative effort originally between the University of Florida (UF) and the Florida Agricultural and Mechanical University (FAMU). The training center’s mission is to train public health workers with special emphasis on rural settings and human immunodeficiency virus/acquired immune deficiency syndrome (HIV/AIDS). A review of this training center’s website, and the 37 others nationwide, reveals that 20 currently have a social media presence using Facebook; 5 of those maintain a Twitter page. Currently, these centers primarily use social media platforms for advertisement of center and community events, as well as offering links to other resources and research for specific areas of specialty.

This study provides an overview of factors common to many organizations related to the implementation of social media. While the term social media is often generally used in reference to Twitter and Facebook, a more specific term for this communication category is social networking sites (SNSs). SNSs are defined as Web-based services that allow individuals to (1) construct a public or semi-public profile within a bounded system, (2) articulate a list of other users with whom they share a connection, and (3) view and transverse their list of connections and those made by others within the system [1]. What makes these sites unique is not only that users can meet people they do not personally know, but that they are able to see and show their network [1].

For the purpose of this study, the focus on SNS centered on Facebook and Twitter due to their popularity. Like various SNSs, Facebook requires individual accounts; users have the ability to modify these accounts, ultimately affecting their potential contacts based on how they create their profile. Twitter is a micro-blogging SNS that has gained popularity in limiting users to write messages in 140 characters or less. Twitter, by allowing users to place a hashtag before a term creating a new searchable topic, has become valuable for professionals who want to network with people in their field and see specific topics regularly updated [2]. While Twitter has only been online since 2006, there is already growing research on its use in public health and by public health organizations [3]. Twitter users have demonstrated use by following health conferences, a developing health story, or learning new sources and Web links for future research [4]. Because of the internal system, using a hashtag, users can highlight specific words in their post that allow for grouping of posts. Thus, users can sort through all posts related to these words, which can help point them in new related directions of searching. The World Health Organization (WHO) used Twitter during a recent health scare, as the hashtag “H1N1” had nearly 12,000 followers [4]. This allowed WHO to update 12,000 people in addition to those to whom the information was forwarded every time there was news to report. Through a May 2013 search, the Centers for Disease Control and Prevention had over 162,000 Twitter followers, and a subgroup at one point had over 400,000 tweets.

Another important theme for one to consider, related to public health workers using SNS, is access. While much research highlights potential applications of SNS in public health, the subject is diminished when considering the access to, or lack thereof, this information to public health workers [5]. Recent budget cuts have halted opportunities for conferences and travel for further educational opportunities [6]. Budget cuts, as well as staff reduction of some county departments in Florida, place stress on the system and communities. Employees are now taking on more responsibilities and tasks beyond their scope of expertise, further reducing their time for training or collaborating with others in the field. These factors, and others, are pushing more formal and informal training online. Pursuing informal professional development through SNS, however, can be difficult as many state health departments, including Florida, block these sites on their Internet filters. The aim of this study was to record the decision-making process for a public health organization as it considered the implementation of social media to advance the center’s goals and mission, by focusing on the perspectives of varying stakeholders within a public health training center, in order to provide considerations for administrators/organizations when deciding to implement a social media presence.

## Methods

This research study was conducted within a case-study model focusing on the RSPHTC’s potential implementation of a social media presence and followed a model of diffusion [7-9]. Rogers’ diffusion model has numerous aspects and perspectives, many of which provided the theoretical framework for this study. The diffusion of innovations theory is one that seeks to explain how, why, and at what rate new technology and ideas spread through a culture [7]. In essence, diffusion can be defined as a process in which a new innovation is communicated through a certain period of time, through various channels, among a social system [7]. Primarily, the diffusion model looks at 4 characteristics related to the diffusion of an innovation: elements of the innovation, types of decisions, the adoption process, and characteristics of adopters.

This project focused on the potential implementation of social media by the RSPHTC. Interviews of various stakeholders provided an account of the variables considered throughout the process. The interview questions were set in an interview guide created through instruments highlighted in the *Interview Guide Approach* [10]. The interview guide was created to more fully understand the knowledge, perceptions, and processes used by the RSPHTC regarding SNS implementation, in accordance with the framework of Rogers’ diffusion of innovations model. A total of 3 interviews were set up with each individual, recorded, and later transcribed verbatim, as described by the *Interview Guide Approach* [10]. The focus of the first interview is getting acquainted, developing rapport, and laying out the area that the researcher would like the interviewee to explore [11,12]. Between the first and second interview, the participant has had time to think more deeply about the experience, and, thus, the second interview is more focused and allows time to explore the experience in depth [11,12]. In the third interview, the researcher asks follow-up questions to fill in and clarify the account of the first 2 interviews, and the participant can add newly remembered information prior to moving on to new information [11,12]. Each interview was conducted face-to-face and recorded by the same individual (MH).

In applying Rogers’ diffusion model to the RSPHTC social media study, the interview questions were grounded thoroughly. The researcher asked questions related to all aspects of the innovation that could be considered by the stakeholders. The focus of these respective interviews with decision stakeholders included their definition and perceptions of social media, determining social media usage in public health, consideration of how social media can affect those within public health, and gauging perceived positive and negative implications for the implementation of social media for the center. Decision makers were chosen as the focus of this study because they are the individuals that would choose if public health workers could use social media or not.

### Recruitment

This study followed the experiences of 15 stakeholders in the RSPHTC’s decision-making process of considering social media implementation. To be interviewed, participants had to meet all three of the following inclusion criteria: (1) being members of the RSPHTC’s management team or advisory board, (2) representing the 2 universities on the training center grant, and (3) being members who were given the responsibility to control day-to-day operations, or consult those making day-to-day decisions for the center (advisory board).

We recruited 15 subjects to participate in this investigation, by personal requests through email. These 15 members represented advisory board members and senior public health researchers and administrators at UF and FAMU. As the RSPHTC is a smaller organization with easily identifiable members prominent in the field of public health and university administration, extra effort was made to hide the specific identities of the participants in this study. In using a singular bound case, however, all thoughts of the SNS decision makers for the center were contained and reflective of the group. Collecting the final responses and sharing them without identifiers allowed the participants to speak more freely on the questions related to SNS implementation, its place within public health, and potential technology blocks imposed at the state and county levels. Each interview was coded into respective themes by trained qualitative researchers (MH & SHT).

### Statistical Analysis

The results section of this study examined the replies of the stakeholders as a whole and separated the interview responses by themes, not by participant.

In total, 15 participants were interviewed three times each, for a total of 45 interviews. Each interview was transcribed and then coded using qualitative methods. To enhance the reliability of the coding process, 2 trained qualitative researchers both coded 3 interviews together to establish an initial code book, and then individually coded the entire set of interviews. At the conclusion of the initial data analysis, the researchers then compared their results, working the data from codes to larger themes.

While there are various methods within qualitative research to analyze data, the format used in this study was the constant comparison method. By using the constant comparison method of analysis, data can be reduced into manageable units and coded information. This method of analysis starts with examining the raw data, looking for key words across all interviews, and grouping segments of the responses into categories. From there, this method can be categorized into 4 states: comparing incidents applicable to each category, integrate categories and their properties, delimiting theory, and finally writing the theory or narrative [10]. What makes this method unique is that it is a continuous growth process, as each stage transforms itself into the next, while previous stages remain in operation throughout the analysis.

## Results

Through the use of qualitative methods, the interviews with the RSPHTC stakeholders revealed six main themes in their responses: (1) personal experience with technology and SNS, (2) use of SNS in public health, (3) use of SNS in work environments, (4) SNS access, (5) ways the RSPHTC could use SNS, and (6) perceived outcomes of SNS usage for the RSPHTC (positive and negative).

###  Personal Experience With Technology and Social Networking Sites

In reviewing the interviews of the RSPHTC decision makers, there was a vast spectrum of personal SNS usage demonstrated. Prior to specifically discussing SNS usage and public health, various questions helped form a baseline of the stakeholders’ collaborative knowledge and usage of technology in general. For the most part, many of the participants in Interview 1, when asked about what technology they use on a daily basis, listed the telephone, their desktop computer, and email as specific programs used. When asked about the technology they use in their job, 2 listed their phone, 4 listed their computer, and all 5 specifically mentioned email. One participant, when asked what program she used most on her computer, stated, “Just tons of email and some Word documents and that kind of stuff, I probably spend 2 hours a day just responding to emails.” Another participant agreed and added, “Each day it takes me until lunch time to respond to all of my emails and phone calls. That is just how people communicate in public health.” Another aspect of technology often mentioned in the interviews related to specific programs used in teaching, as many of the RSPHTC stakeholders and administrators are also professors.

One stakeholder interviewed seemed to be very passive about SNS in general and made numerous comments about the time needed to maintain a regular presence. Different than others interviewed, who often spoke on how using SNS helped them make connections and allowed them to communicate with others on their schedule, this stakeholder expressed that participating in SNS could be work and almost feel like “an obligation.” This participant, when asked about personal SNS usage, stated “well, that’s an interesting question because everybody around me uses them and they friend me, or they link me.” She continued by clarifying her usage as, “I’m like 90% passive in my use because I just don’t have the time to be active. It also does not really fit my personality to be actively putting things out there about myself.” Ultimately this public health professional seemed content with the status of her friendships and how she communicates with the people in her life and primarily saw SNS as a barrier to friendships and not as a tool to make these connections stronger or more convenient.

In sum, the questions from Interview 1 that probed how the RSPHTC stakeholders use SNS in their personal life resulted in a varied mix of responses showing their collaborative commitment and indifference. Several people interviewed seemed to rely on SNS as a way to stay in touch with friends, past and present. Other people who were interviewed did not seem to be motivated to try and keep up with this growing social medium, content with how they currently communicate with others. Another set of comments, which revealed itself within this theme, related to participant questions on what exactly quantifies participation. Several of the stakeholders offered that they sometimes go onto SNS to look at pictures, see what friends and relatives were doing, but often not posting themselves or accepting the friend requests proposed. It can be said, however, that the RSPHTC decision makers all seemed familiar with SNS and their features. Furthermore, most were able to identify specific SNS by name, most citing Facebook, Twitter, and LinkedIn.

###  Use of Social Networking Sites in Public Health

In examining the responses of the RSPHTC workers from Interview 2, with regard to how they use SNS at work and within the field of public health, the answers were often difficult to distinguish between general public health and their personal work advancement. For the most part, many of the responses aligned to the stakeholders sharing how they make connections in the field of public health or their field of academia with SNS versus how to use, or teach how to use, the tools to directly help public health initiatives. The majority of answers described usage as participating in alumni groups, networking with known others who have similar sub-interests in public health, and overall professional development. There also seemed to be a connection of usage in a professional context, with personal usage, as those who incorporated SNS use into their daily lives with friends and family tended to be more determined to implement SNS for the center. The need to keep up with professional development, however, seemed to be a motivator for all, even for those who had previously answered personal use questions with responses indicating their natural tendency to be passive or indifferent to SNS usage. Primarily responses from Interview 2 could be broken down into people describing how they use SNS in the field and ultimately how it compares to the current methods of public health workers’ professional development.

In addition to using SNS as a means to communicate with students and known colleagues, the RSPHTC stakeholders also addressed how they perceive these tools as a forum to interact with others in the field. Most (13/15) of the interviewees stated that they had never followed or tried to connect with someone in the field of public health that they did not already know personally. When wanting to see the work of another professional in the field, for the most part, they would just research their efforts on Google, read about new ideas in academic journals, or watch YouTube and webinar presentations. None of the participants had used a microblogging site, like Twitter, to follow an unknown person in public health. The concept of “following” an unknown person, on a professional or personal level, seemed to be a large deterrent for using a site like Twitter by everyone interviewed. Only 1 person questioned actually used Twitter, but she claimed to deliberately avoid public health discussions and professionals and follow only personal friends. She clarified by stating “When I’m on Twitter, that’s kind of like my time. I’m really not focused on work or public health issues so to speak…I mean public health is part of my life all day long, but when I’m on Twitter at night I’m trying to kind of relax.”

###  Use of Social Networking Sites in Work Environments

As the discussion turned from describing the general role of SNS in the field of public health toward how the RSPHTC could specifically use these sites to help public health workers, a theme of general work environment usage of SNS emerged. Most of the stakeholders interviewed, prior to considering how the RSPHTC could use these tools, shared their thoughts on the access, or lack of access, public health workers have to SNS. Many discussed the imposed block of SNSs through the state (Florida) public health network, which provides Internet access to county health departments, as a way to flush out their thoughts on how the RSPHTC might approach SNS implementation. Having limited access to the intended target audience during their work time seemed to cause a wide spectrum of thoughts on the value of creating an SNS presence. This discussion also, for several interviewed, touched on the subject of smartphones and their role during the workday for public health employees.

###  Social Networking Site Access

While some interviewed commented on how they did not want to discuss the state’s ban of SNSs, often because they did not know enough of why the ban was there to begin with, others were willing to talk about it directly. One stakeholder interviewed made her opinions clear by saying “I think it should be lifted because I do think, more and more, there’s a lot of good information that’s out there on social media.” She went on to elaborate that the ban “makes it tough on public health workers to stay up-to-date, as many do not want to look at these sites once they get home.” This comment continued an ongoing theme others had mentioned in various ways, on questioning the interest level of public health workers to use their own time to use SNS in a professional way. Another person interviewed explained “public health workers are not like teachers, where their classroom is their domain, they just do what the state, or county tells them.” Several others interviewed also alluded to the fact that many public health workers do not have the freedom to do things “their way” but rather need to stick with a scripted response so the public is often hearing 1 universal message.

As the discussion on whether state or county public health workers should have access to SNS during the workday continued, the perceived pros and cons emerged. One person interviewed stressed that getting information through SNS is in line with what people use every day. She stated “I think it’s good technology, technology people are used to, and so it’s really important they should be able to access information in that way.” This same participant continued with her thoughts on potentially using SNS as a message board by public health workers willing to collaborate, or internally for a public health organization as a whole. She explained “these sites save time in the day so if you can just use them, go back and forth and have that collaboration, it makes it easier than trying to get a meeting together.”

A tangential issue to access of SNS while at work, which was repeated by the majority of RSPHTC stakeholders interviewed, was the use of smartphones by employees. For many, the net outcome of this way of using the SNS (blocked computer for work and a smartphone for personal usage) resulted in not allowing for the positive aspects of SNS usage, while also not removing the potential negative aspects of SNS use by employees. One person explained that she used her smartphone at work on a “limited basis.” She said that she used the phone “sometimes for work, where I text people, or look up something in a meeting.” She also added, however, that “sometimes I use it to check my Facebook, personal email.” When asked how proportionate the times are between personal and professional usage, she laughed and said “oh, probably 80% personal.” When asked another follow-up question for her opinion of this usage related to work efficiency, she said “Yeah, I do not know if I would like it for the people who work for me, to do the same but I guess they do.” She continued by adding “it is just the way it is, we now all have supercomputers in our pockets.”

###  Ways the Rural South Public Health Training Center Could Use Social Networking Sites

When compiling the interview responses from Interview 3, which directly paired SNS and the RSPHTC, the vast majority of comments reflected that the RSPHTC stakeholders see this tool as a means for marketing and showcasing center activities. Currently, the website displays announcements of new educational sessions, courses, deadlines, and upcoming events. This website, however, does not have the ability to pop up new information on a stream like Facebook or Twitter, where people get newly updated information. To currently obtain the center’s announcements and marketing efforts, public health workers and community members would have to actively choose to check the website. The website does, however, have a feature allowing for people to sign up for email messages when new posts are made; however, to date no one has signed up for this feature.

Prior to describing perceived positive and negative outcomes for SNS usage, many also touched on logistical issues related to resources needed to properly manage SNS. When considering how the RSPHTC could set up SNS for long-term success, a great disparity arose between the 2 sites most referenced, Facebook and Twitter. Facebook, for many, was a tool that could be more controlled and would allow for attention given to a smaller group of people who are more closely affiliated with the center. Twitter, however, was seen as a tool that could potentially introduce the center to a larger audience but required a much more active participation and commitment. The difference of opinion for these 2 sites also reflected differences within the group on whether the mission of the center was to primarily focus on the regional aspect of training Florida public health workers with HIV/AIDS education or in its larger role as a national training center available to anyone online.

### Perceived Outcomes of Social Network Site Usage for the Rural South Public Health Training Center

As RSPHTC stakeholders considered the impact of SNS usage (see Table 1) in Interview 3, the majority of responses continued to reflect the positive themes of marketing opportunities as well as using those efforts to reach certain younger demographics. A third and fourth subtheme that surfaced reflected the stakeholders’ positive impressions of the cost and ease of use of these sites. Finally, and usually only when asked specifically, some people interviewed considered the SNS that the RSPHTC could construct as a platform for public health workers to collaborate with one another in a formal and informal way.

While the RSPHTC decision makers were able to list several potential positive outcomes for tangible SNS usage now, or in the future, the responses for possible negative outcomes often reflected the fear of the unknown. Answers that reflected a specific negative condition created by SNS usage were rare; however, many of those interviewed seemed very concerned that perhaps they were missing an angle they had not considered. Supporting this mind-set, many people during their responses to these questions made mention of wanting to educate themselves more on how other training centers are using this model. Furthermore, there seemed to be a cost analysis for many in considering how many positive occurrences there need to be to balance out a potential negative occurrence that would reflect badly on the center or the 2 sponsoring universities. In the end, the set of questions from Interview 3 asking the RSPHTC decision makers to differentiate between the positive and negative outcomes resulted in more tangible responses for positive outcomes and a smaller sample of often fear-based answers for possible negative ones. This is not to say, however, there were no identifiable negative outcomes, as some mentioned concerns related to time, resources, inappropriate comments, and privacy.

**Table 1:** Most frequently mentioned positive and negative outcomes of SNS implementation perceived by RSPHTC stakeholders positive.

Positive	Negative
Marketing opportunities, for both public health workers and community members	Privacy
Reaching younger demographics	Dedicated resources for managing
Low cost	Stigma (related to HIV/AIDS focus)
Ease of use	Low level of discourse
Act as website portal	
Facilitate collaboration between public health workers or patient groups	

## Discussion

### Principal Results

As stated throughout this study, the opportunity to research an ongoing decision-making process, of very accomplished and distinguished public health professionals, is one many people within and outside the field of public health can learn from. Organizations that are not as well staffed, do not implement SNS usage, or do not have the experience of these professionals interviewed can be given a head start on the background of this innovation, as well as a long list of potential positive and negative ramifications to consider. For those who are advocates of social media, the worries of those who have not chosen to implement this for their organization can also be helpful as they can see perceived barriers whether they are accurate or not. This study, whether read from the perspective of the public health worker, administrator, or someone at the state health department level, illuminates issues that have not often been discussed beyond personal conversations. To date, however, the RSPHTC has chosen not to implement a social media presence, due to a lack of resources from cutbacks in the grant money funding the project.

### Comparison With Prior Work

Public health organizations are slowly beginning to adopt the use of SNS. Analysis of SNS use shows that health departments and other public health organizations mainly use SNS as a way to disseminate health tips and information as well as information about specific organizations and events [13-18]. Unlike the current research, this study goes beyond the analysis of types of messages used on SNS and examines public health organizations’ decision-making processes on whether or not to adopt SNS.

### Limitations

The study examined only the SNS decision-making process at a single organization (RSPHTC); however, these 15 individuals are highly accomplished and experienced in their fields, making these results more broadly generalizable.

### Conclusions

From the researchers’ experience through all their conversations with individuals in public health, RSPHTC stakeholders, managers from counties throughout Florida, and those in the state health department who gave their time and official comments, there seems to be a lack of motivation and forum to have an open discussion on SNS implementation in the field of public health. Blocking aspects of the Internet for professional working adults, charged with protecting the public well-being of our entire society, is a significant philosophical issue. While removing the filter could potentially allow for computer viruses through the network, or employees making comments that reflect poorly on an organization, the fact is that many other fields and organizations have found ways to deal with these similar obstacles. However, pushing for more ways to incorporate SNS in the field of public health would not be as necessary if the majority did not see a need or did not want implementation. Having said that, the majority of people we interviewed seemed excited about the potential applications and often characterized the use of SNS as inevitable, making the lack of motivation for discussions moving the process along unclear. While there appears to be an environment of people discussing the Internet block among themselves, there seems to be a lack of open discussion with those at the state level. At this point, the potential implementation of more SNS use in public health stands at a tipping point, full of possible ways of application, further collaboration, and marketing and educational opportunities, but it lacks a singular voice with the ability to move the debate toward new policy. Hopefully, this study can create a foundation for many in the field to understand the current state of SNS in the field, as well as give a composite overview of many of the varied mind-sets on this innovation.

## Abbreviations

FAMU: Florida Agricultural and Mechanical University
RSPHTC: Rural South Public Health Training Center
SNS: social networking sites
UF: University of Florida
